# Macular pigment density changes in central serous chorioretinopathy

**Published:** 2018

**Authors:** Ruxandra Tudosescu, Cristina Mihaela Alexandrescu, Sinziana Luminita Istrate, Alexandra Vrapciu, Radu Constantin Ciuluvica, Liliana Mary Voinea

**Affiliations:** * Ophthalmology Department, Regina Maria Private Health Care Network, Dorobanti Hyperclinic, Bucharest, Romania; ** Ophthalmology Department, Bucharest University Emergency Hospital, Bucharest, Romania; *** Ophthalmology Department, “Carol Davila” University of Medicine and Pharmacy, Bucharest, Romania; **** Division of Anatomy, Faculty of Dental Medicine, “Carol Davila” University of Medicine and Pharmacy Bucharest, Romania

**Keywords:** macular pigment optical density, central serous chorioretinopathy

## Abstract

**Aim:** To present a series of 2 cases of central serous chorioretinopathy and the changes in the macular pigment optical density during the evolution of the disease.

**Material and methods:** A 32-year-old patient presented himself for blurred vision on his LE. The SD OCT imaging revealed serous macular detachment of the neurosensory retina on the LE. The MPOD results were 0.72 on RE and 0.91 on LE. After treatment and resorption of the subretinal fluid, the MPOD values were 0.72 on the RE and 0.82 on the LE. The second patient was a 36-year-old male with metamorphopsia on LE and serous macular detachment on this eye. The MPOD results were 0.43 on RE and 0.58 on the LE and, after treatment, they were 0.38 on the RE and 0.43 on the LE.

**Conclusions:** Central serous chorioretinopathy is a disease of unknown pathophysiology in which we observed a higher MPOD on the eye with CSC than on the fellow eye and a decrease in the MPOD value after the resorption of the subretinal fluid.

**Abbreviations:** L = lutein, Z = zeaxantin, MZ = mezozeaxantin, AMD = age related macular degeneration, MPOD = macular pigment optical density, MP = macular pigment, HFP = Heterochromatic Flicker Photometry, CSC = central serous chorioretinopathy, RE = right eye, LE = left eye

## Introduction

Central serous chorioretinopathy (CSC) is an idiopathic posterior pole disease which is characterized by the development of a detachment of the neurosensory retina that can also associate a retinal pigment epithelium (RPE) detachment [**1**]. The pathophysiology of this disease is controversial and there are theories that revolve around the role of the choroid in this disease [**2**]. New imaging like swept source spectral-domain optical coherence tomography (SD-OCT) with enhance depth imaging (EDI), and angio SD-OCT are trying to bring new evidence regarding the mechanisms of the CSC and maybe new treatment options [**3**]. One of the new hypothesis is the mineralocorticoid pathway and the aggravation of fluid accumulation in diseases that involve high levels of glucocorticoids - like Cushing syndrome or corticosteroid treatment [**4**].

Multimodal imaging is the best approach in the follow-up and guided treatment of this disease and includes SD-OCT, angiofluorography - for the leakage point, indocyanine green angiography - for the changes in the choroid and fundus photography. 

The macular pigment (MP) consists of the natural xanthophyll pigments Lutein (L), meso-zeaxanthin (MZ) and zeaxanthin (Z). The major roles of the MP are the protection against age related macular degeneration (AMD), the antioxidant role against the oxidative stress that can be induced by free oxygen radicals and visual performance improvement (which includes glare and contrast sensitivity) [**5**-**9**]. Thus, the photoreceptors that are located in the macula, receive a natural protection. 

The macular pigment is located primarily in the inner plexiform layer and the photoreceptor axon layer of the macula [**10**].

## Material and methods – case series reports

We present a series of 2 cases of patients with acute CSC.

**Fig. 1:**
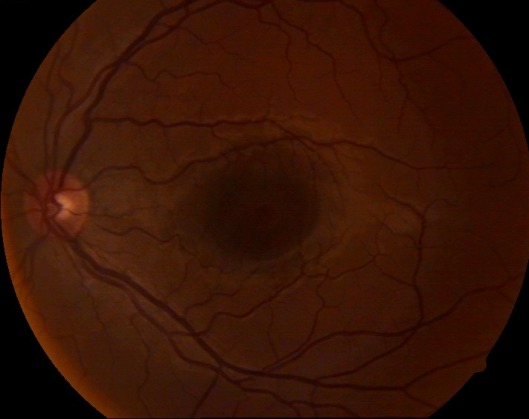
Left eye – serous macular detachment (case 1)

***CASE 1:*** A 32-year-old patient presented himself in the Ophthalmology Clinic at the University Emergency Hospital, Bucharest for blurred vision on his left eye (LE) for 2 weeks. On admission, the ophthalmological examination showed best-corrected visual acuity (BCVA) on RE 1 and difficulty on the LE 1. Slit lamp examination and aplanotonometry were within normal limits on both eyes. Fundus examination on the RE was normal and on the LE revealed serous macular detachment of approximately 2 papillary diameters (**Fig. 1**).

**Fig. 2,3:**
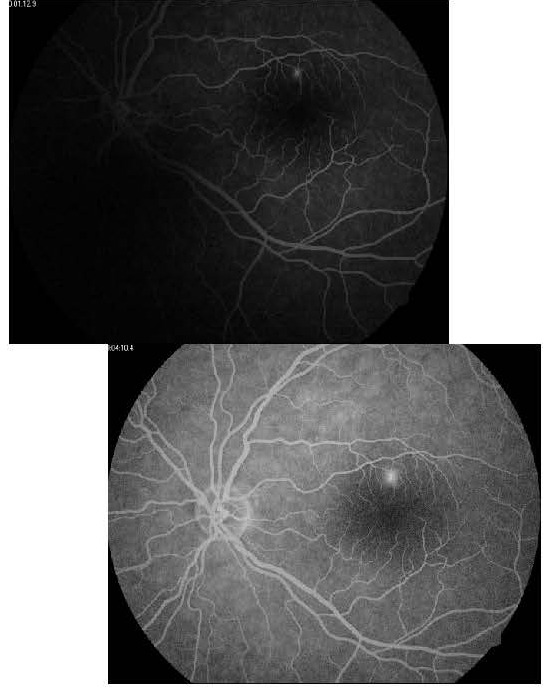
LE - Early and late phase of the angiofluorography with the expansile dot pattern (case 1)

The angiofluorography showed a hyperfluorescent dot that grew in size and intensity in the late phases of the angiography (**Fig. 2,3**).

**Fig. 4:**
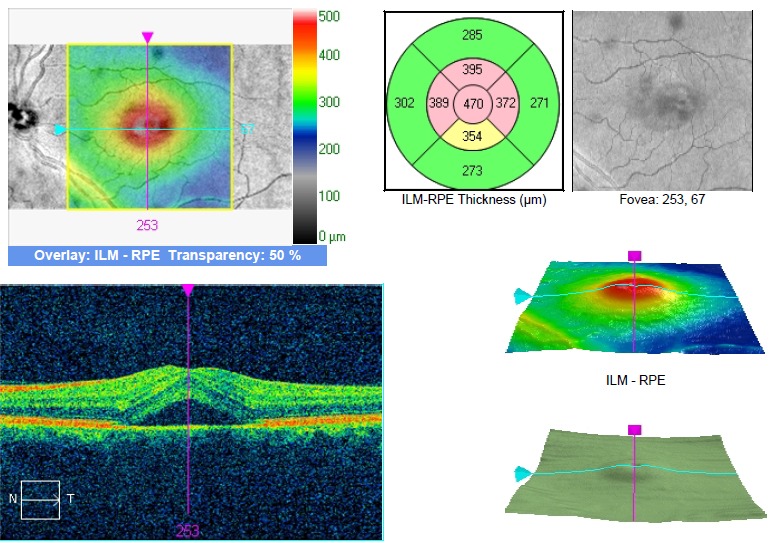
OCT of the LE with serous detachment of the neuroretina (case 1)

The SD OCT imaging revealed the presence on the LE of serous macular detachment of the neurosensory retina with a central thickness of 470 microns. The central thickness of the macula on the RE was 296 microns (**Fig. 4**).

Glare sensitivity was measured and the results were 9 out of 10 on the RE and 4 out of 10 on the LE, while contrast sensitivity was 9 out of 10 on RE and 6 out of 10 on LE.

**Fig. 5:**
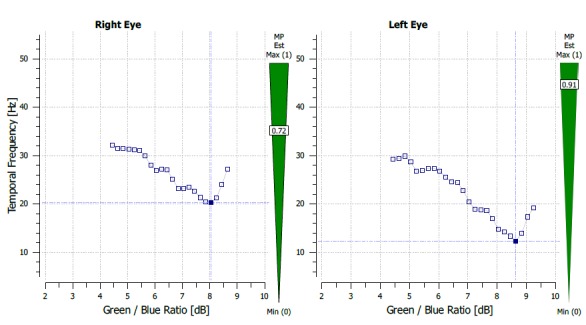
MPOD measurement report before treatment (case 1)

The Macular Pigment Optical Density (MPOD) was evaluated using Macular Pigment Screener MPS II (Electron eye technology, Cambridge, United Kingdom). The MPOD results were values that ranged on a scale of 0-1. The results were 0.72 on the RE and 0.91 on the LE (**Fig. 5**).

In this context, the diagnosis of LE central serous chorioretinopathy was established and treatment was started with oral acetazolamide arena 500 mg three times per day, aspacardin 100 mg three times per day, NSAID daily and topical NSAID (bromfenac) three times per day. After 8 months of treatment and monthly follow–up, the complete resorption of the subretinal fluid was achieved.

**Fig. 6:**
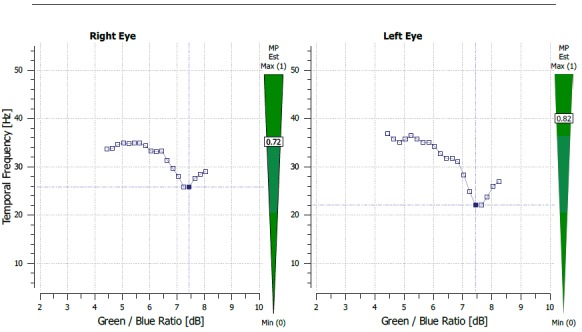
MPOD measurement after treatment and with no subretinal fluid (case1)

**Fig. 7:**
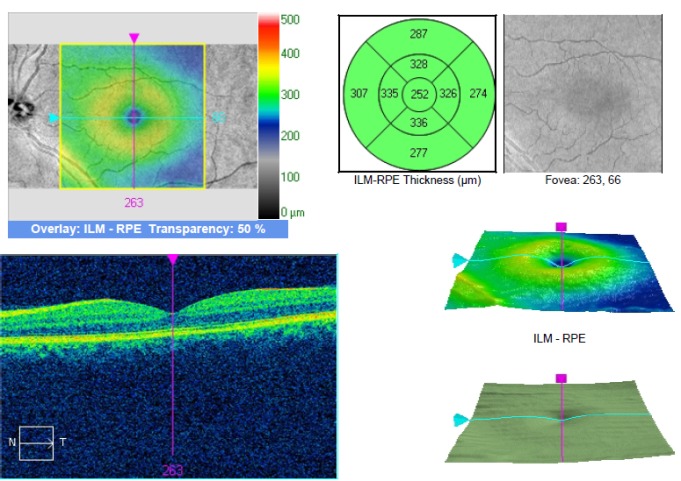
SD OCT of the LE after treatment (case 1)

The MPOD values were 0.72 on the RE and 0.82 on the LE and, the central macular thickness of the LE was 252 microns on SD–OCT (**Fig. 6**,**7**).

***CASE 2:*** The second patient was a 36-year-old male who presented himself in our clinic for metamorphopsia on his LE for 1 month. Clinical evaluation on admission showed a BCVA 1 on the RE and 0.7 on the LE. Slit lamp examination and IOP were normal. Fundus examination revealed serous macular detachment on the LE and a normal one on the RE. 

**Fig. 8:**
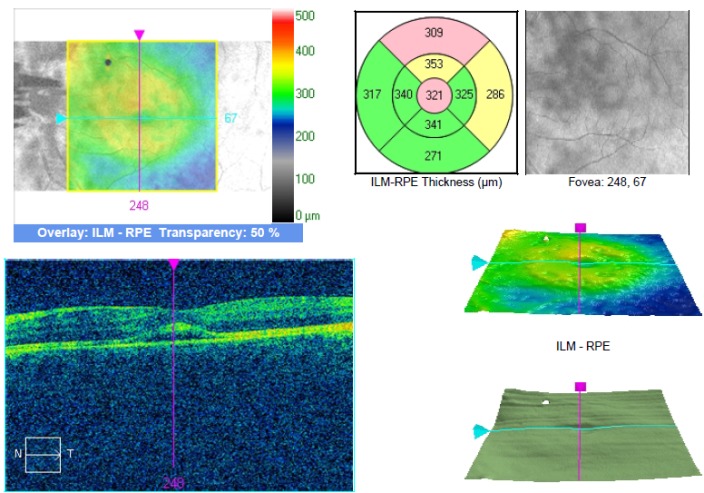
SD OCT of the LE with neurosensory detachment of the retina (case 2)

SD OCT imaging showed the presence of serous macular detachment of the neurosensory retina with a central thickness of 321 microns on the LE. The central thickness of the macula was 271 microns on the RE (**Fig. 8**).

Measured glare sensitivity was 9 out of 10 on the RE and 6 out of 10 on the LE, while contrast sensitivity was 9 out of 10 on the RE and 6 out of 10 on the LE.

The MPOD results were 0.43 on the RE and 0.58 on the LE.

Treatment was initiated with oral acetazolamide arena 500 mg twice per day, aspacardin 100 mg twice per day, NSAID daily and topical NSAID (bromfenac) in the LE three times per day.

After 2 months of treatment, we achieved the resolution of the subretinal fluid. 

**Fig. 9:**
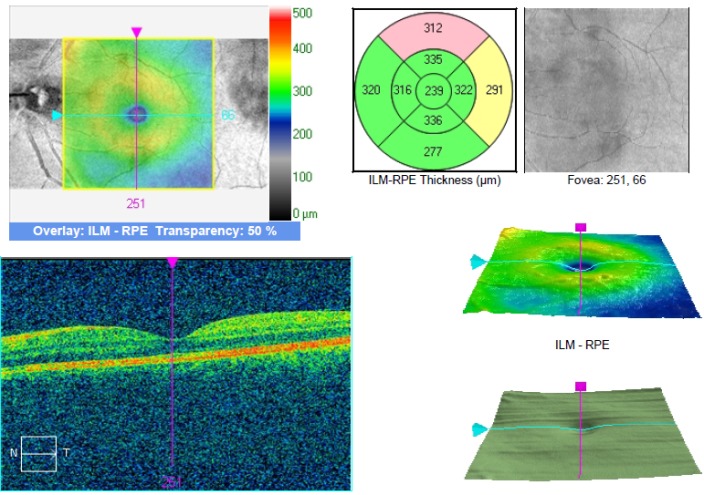
SD OCT of the left eye after resorption of the subretinal fluid (case 2)

**Fig. 10:**
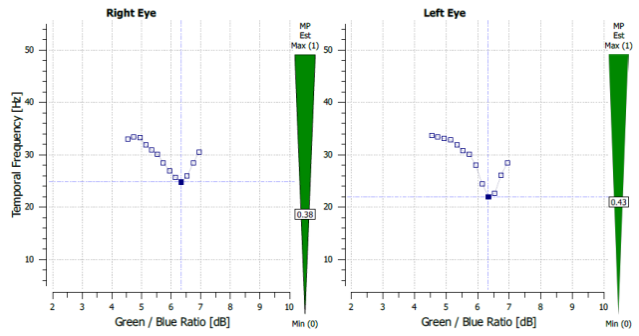
MPOD measurement after treatment and no subretinal fluid (case 2)

The central retinal thickness on the LE was 239 microns, with a BCVA of 1 on the LE, a glare and contrast sensitivity of 9 out of 10 (**Fig. 9**). THE MPOD values were 0.38 on the RE and 0.43 on the LE (**Fig. 10**). 

## Discussion

Current literature does not have extensive studies regarding MPOD and CSC. Putnam et al. tried to measure the MPOD and did a full spatial mapping with a new, customized heterochromatic flicker photometer in a patient with resolved CSC. They showed that there might be some asymmetry after the resolution of the disease [**11**].

Another study from Sasamoto et al. (2010) tried to measure the MPOD in acute and chronic CSC comparing it with the fellow eyes. They showed that in chronic CRSC, MPOD may decrease, thus the MP density being an indicator for the chronic form of the disease [**12**].

After analyzing the 2 cases we observed that MPOD was higher on the eye with CSC than on the fellow eye, with a decrease after the resolution of the sub retinal fluid. 

The method used in the measurement of the MPOD was heterochromatic flicker photometry (HFP). Through this technique, the test stimulus, which can be in the shape of a disk or ring, alternates 2 wavelengths: one blue at 460 nm - absorbed by the macular xanthophylls and one green at 540 nm that is not absorbed. This test requires that a patient has a good visual acuity in order for him to fixate the target and proper training and attention [**13**]. We hypothesize that the higher MPOD value might be due to the presence of the fluid in the intraretinal space and the optical changes that it causes. The presence of the subretinal fluid probably also causes, besides a hypermetropic shift and a detachment of the photoreceptors from de RPE, a change in the perception of the test stimulus, thus generating the higher MPOD value, HPF being a method that uses the absorbance of light at different wavelengths. We observed that the MPOD in both cases was lower after treatment of the eye with CSC (case 1 – 0.91 before, 0.82 after, with a difference of 0.19 before and 0.1 after between the 2 eyes; case 2 - 0.58 before and 0.43 after, with a difference of 0.15 before and 0.05 after). The data also showed a decrease in the interocular difference of the MPOD. 

The 2 cases showed a pattern in the MPOD changes, so a larger study with a bigger sample size should be conducted to see the reproducibility of the data. From our knowledge, there is no other study that has shown the same changes or that has studied the MPOD before and after the resorption of the subretinal fluid. 

## Conclusions

Central serous chorioretinopathy is a disease with unknown pathophysiology. Current studies are now trying to find more information regarding the changes in the choroid or the mineralocorticoid pathways. The macular pigment is located in the foveal zone where the subretinal fluid accumulates more frequently, so the changes in the MPOD that we observed could be due to changes in perception of the stimulus, showing a higher value of the MPOD that decreases after the fluid is resorbed. 

**Acknowledgements**

1. AMD Nobel Pharmaceutical is gratefully acknowledged for all the support offered in data collection. 

2. All the authors have equally contributed to this study.

**Disclosures**

None
